# Supplementary material for: Impact of Body Composition Parameters on Lung Function in Athletes
Source: Nutrients. 2022 Sep 16;14(18):3844. doi: 10.3390/nu14183844 (PMC9500777; doi:10.3390/nu14183844)
Supplement: Supplementary file 1 [file nutrients-14-03844-s001.zip › nutrients-1876622-supplementary.pdf]

Supplemental material Table S1. Sports disciplines in male and female athletes

Sport Discipline	Male (335)	Female
Basket	3 (0.9)	2 (2)
Boxing	6 (1.79)	1 (1)
Cycling	37 (11.04)	1 (1)
Football	66 (19.7)	1 ()
Karate	10 (2.99)	2 (2)
Long distance running	89 (26.57)	22 (22)
Mid distance running	31 (9.25)	18 (18)
Rugby	1 (0.3)	21 (21)
Short distance running	20 (5.97)	14 (14)
Speed walking	4 (1.19)	-
Style dancing	3 (0.9)	14 (14)
Swimming	29 (8.66)	13 (13)
Tennis	23 (6.87)	4 (4)
Triathlon	12 (3.58)	1 (1)
Volley	1 (0.3)	1 (1)

Data are expressed as number and percentage.

Supplemental material Table S2. Exercise intensity and spirometry parameters in all population.

Variable	Moderate (n=245)	High Intensity (n=190)	p-value
FVC	4.45 ±1.03	4.97 ± 0.89	<,=0.0001
FEV1	3.8 ±0.7	4.2 ±0.9	<,=0.0001
FVC/FEV1	85.2 ±9.1	84.1 ±9.3	0.22

Supplemental material Table S3. Exercise Intensity and spirometry parameters in males.

Variable	Moderate (n=166)	High Intensity (n=167)	p-value
FVC	4.8 ±0.95	5.1 ± 0.95	0.01
FEV1	4.1 ±0.86	4.2 ±0.65	0.07
FVC/FEV1	84.2 ±9.4	83.9 ±9.3	0.78

Supplemental material Table S4. Exercise Intensity and spirometry parameters in females.

Variable	Moderate (n=79)	High Intensity (n=21)	p-value
FVC	3.6 ±0.59	3.9 ± 0.49	0.03
FEV1	3.2 ±0.59	3.3 ±0.5	0.27
FVC/FEV1	87.2 ±8.1	85.2 ±9.4	0.33

FEV1: forced expiratory volume during 1 s; FVC: forced vital capacity

Supplemental material Table S5. Correlation between body composition and spirometry parameters.

Variable	FVC	FEV1	FVC/FEV1
FFM	0.55	0.49	-0.11
FFM%	0.24	0.25	-0.06
MM	0.70	0.64	-0.11
MM%	0.41	0.43	0.07
FM	-0.13	-0.18	-0.11
FM%	-0.34	-0.37	-0.06
WC	0.29	0.19	-0.21
WHR	-0.03	-0.13	-0.19
ABSI	0.07	0.03	-0.08

FFM: fat-free mass; FM: fat mass; MM: muscle mass; FEV1: forced expiratory volume during 1 s; FVC: forced vital capacity; WC: waist circumference; WHR: waist-to-height ratio;

Supplemental material Table S6. Multivariate analysis model 2

	Male			Female		
Variable	Coefficient	p-value	R2	Coefficient	p-value	R2
FEV1						
FFM	0.16	<,= 0.0001	0.35	0.30	0.013	0.37
MM	0.45	<,= 0.0001	0.44	0.34	0.004	0.38
FVC						
FFM	0.03	0.8	0.20	0.26	<,=0.0001	0.23
MM	0.67	<,=0.0001	0.38	0.46	0.001	0.30

FFM: fat-free mass; MM: muscle mass; FEV1: forced expiratory volume during 1 s; FVC: forced vital capacity; Multivariable regression analysis corrected for age, BMI, sport intensity moderate vs high and endurance discipline, and smoking habit.

Supplemental material Table S7. Multivariate analysis model 3

	Male			Female		
Variable	Coefficient	p-value	R2	Coefficient	p-value	R2
FEV1						
FFM	0.02	0.60	0.46	0.12	0.29	0.44
MM	0.16	0.01	0.47	0.15	0.176	0.44
FVC						
FFM	0.02	0.745	0.47	0.32	0.014	0.33
MM	0.32	<,=0.0001	0.0.41	0.27	0.032	0.32

FFM: fat-free mass; MM: muscle mass; FEV1: forced expiratory volume during 1 s; FVC: forced vital capacity; Multivariable regression analysis corrected for age, height, endurance discipline, and smoking habit.
